# 1869—1902. State Medical Association of Texas

**Published:** 1902-05

**Authors:** 


### THE TEXAS MEDICAL JOURNAL.

ESTABLISHED JULY, 1885.

PUBLISHED MONTHLY.—SUBSCRIPTION $1.00 A YEAR.

VOL. XVII. AUSTIN, MAY, 1902. No. 11.

### 1869—1902.

### STATE MEDICAL ASSOCIATION OF TEXAS.

**Preliminary Announcement and Program of the Thirty-fourth Annual Meeting, Dallas, Texas, Tuesday, Wednesday, Thursday and Friday, May 6, 7, 8 and 9, 1902.**

### COMMITTEE ON ARRANGEMENTS.

JOHN O. MCREYNOLDS, Chairman.
R. W. BAIRD.
J. B. SMOOT,
J. B. SHELMIRE.
J. H. FLORENCE.
H. L. MCNEW.
E. DUNLOP.

### OFFICERS.

TAYLOR HUDSON, Belton, *President.*
S. C. RED, Houston, *First Vice-President.*
J. W. NIXON, Gonzales, *Second Vice-President.*
W. A. WATKINS, Kemp, *Third Vice-President.*
H. A. WEST, Galveston, *Secretary.*
R. F. MILLER, Sherman, *Treasurer.*
W. H. MOORE, Runge, *Orator.*

### OFFICERS OF SECTIONS.

1. *General Medicine*—J. W. Scott, Houston, Chairman; S. E. Hudson, Austin, Secretary.
2. *Obstetrics and Diseases of Children*—J. M. Frazier, Belton, Chairman; W. C. Blalock, Kosse, Secretary.
3. *Surgery*—A. C. Scott, Temple, Chairman; A. C. McDaniel, San Antonio, Secretary.
4. *Medical Jurisprudence*—M. L. Graves, San Antonio, Chairman; Geo. H. Lee, Galveston, Secretary.
5. *State Medicine*—W. S. Carter, Galveston, Chairman; G. T. Thomas, Rogers, Secretary.
6. *Gynecology*—J. T. Feild, Fort Worth, Chairman; C. M. Alexander, Coleman, Secretary.
7. *Ophthalmology, etc.*—R. E. Moss, San Antonio, Chairman; H. L. Hilgartner, Austin, Secretary.
8. *Dermatology*—Geo. H. Lee, Galveston, Chairman; F. E. Daniel, Austin, Secretary.
9. *Pathology*—A. J. Smith, Galveston, Chairman; Wm. Gammon, Galveston, Secretary.

### TRANSPORTATION.

All railroads in the State offer the following rates: Tickets will be sold to Dallas and return on the convention plan rate. providing for distances taking a higher rate than $3.00 one way, the round trip rate will be one fare plus 10 per cent. For points taking a less rate than $3.00, for the round trip the fare will be one and a third. Tickets to be on sale May 5th, return limit May 10th.

### HOTEL RATES.

Oriental, $2.50 to $3.00 per day.
St. George, $1.50 to $2.50 per day.
McLeod, 75 cents to $1.00 per day.

### MEMORANDA.

1. Papers here announced will take precedence of those subsequently offered.
2. Papers will be read in the order as appears upon the program.
3. Papers read before the Association must be handed to the Secretary as soon as they shall have been read. Such papers, as well as those read by title, belong to the Association, but authors, if they so desire, may publish them elsewhere prior to publication in the Transactions.

4. Alterations will not be made in a paper after it is in type, except at the expense of the author.

5. Illustrations will be inserted without expense to author.

6. Papers read shall not occupy more than twenty minutes, except by permission. Discussion shall be limited to five minutes, unless the time be extended by unanimous consent of the sections, except as to the author of a paper, who shall have ten minutes in which to close.

No member shall speak more than once to the same subject without permission. (See By-Laws, Art. XV.)

---

### Preliminary Program.

The session will begin Tuesday morning at 11 o'clock. The place of meeting, the City Hall. Members and applicants are requested to assemble early in order to register, as far as possible, before the opening exercises. The Secretary, Treasurer and a special committee will be present to aid registration. Applicants for membership who belong to district or county societies in affiliation with the State Association may join upon certificate duly signed and accompanied with $5.00, one year's dues, and $2.50 initiation fee. Other applicants will sign blank form, giving postoffice address and county. Each application must have the signature of two members and be accompanied by $7.50, covering one year's dues and the initiation fee. Such applicant must furnish diploma or other documentary evidence of graduation for inspection by the Judicial Council. Badges will be furnished members and applicants after registration.

In accordance with a resolution adopted at San Antonio, requiring that the time for the meeting of the several sections to be printed in the program, and that all papers in a certain section be presented in their regular order at the time specified, the following is submitted:

FIRST DAY—TUESDAY, MAY 6, 11 A. M.

Call to order by chairman Committee of Arrangements, Dr. John O. McReynolds, Dallas.

Invocation, Dean Hudson Stuck.

Welcome, by Mayor Ben E. Cabell.

Welcome, representing the medical profession of Dallas, Dr. J. H. Smart.

Response, Dr. Taylor Hudson, President.

Roll call.

Reading minutes.

Secretary's Annual Report, Dr. H. A. West, Galveston.

Treasurer's Annual Report, Dr. R. F. Miller, Sherman.

President's Annual Message and Recommendations, Dr. Taylor Hudson, Belton.

Appointment of Provisional House of Delegates to be composed of representatives from affiliated societies to consider and report upon revision of Constitution and By-Laws.

*Afternoon Session, 2 o'clock.*

Medical Section.

*Night Session, 8 o'clock.*

Section on Obstetrics and Diseases of Children.

SECOND DAY—WEDNESDAY, 9 A. M.

Executive business.

Surgical Section.

*Afternoon Session.*

Section on Medical Jurisprudence, 2 to 4:30 o'clock.

Memorial services, 4:30 to 6 o'clock.

*Night Session, 8 o'clock.*

Section on State Medicine.

THIRD DAY—THURSDAY, 8 A. M.

*Morning Session.*

Executive business, 8-9.

Section on Ophthalmology, 9-10.

Section on Pathology, 10-12:30.

*Afternoon Session, 2 o'clock.*

Executive business.

Appointment of Nominating Committee.

Recall of sections in rotation.

*Night Session 8 o'clock, sharp.*

President's Address, Dr. Taylor Hudson.
Annual Oration, Dr. W. H. Moore.
Reception, 9 o'clock.

FOURTH DAY—FRIDAY, 9 A. M.

Report of Nominating Committee.
Election of officers.
Unread papers.
Executive business.

### SECTION ON GENERAL MEDICINE.

1. Chairman's Address. J. W. Scott, Houston.
2. "Therapeutics of the Glycero Phosphates." H. A. West, Galveston.
3. "The Poisonous Snakes and Spiders of Texas." H. W. Crouse, Victoria.

*Symposium on Pneumonia.*

4. "Pathology." R. W. Knox, Houston.
5. "Etiology and Symptomatology." S. C. Red, Houston.
6. "Treatment." R. T. Morris, Houston.
7. "The Origin of Sensation and Thought from an Embryological Standpoint." J. M. Fort, Paris.
8. "A Comparative Study of the Value of Methylene Blue and Quinine in the Treatment of Malarial Fever." John T. Moore, Galveston.
9. "Achlorohydria Gastrica." J. W. McLaughlin and S. M. Morris, Galveston.
10. "Influenza." J. R. Nichols, Terrell.
11. "The Deaf-Blind and What Texas Does for Their Education." M. M. Smith, Austin.
12. "The Action and Uses of Digitalis." S. E. Hudson, Austin.
13. "Typhoid Fever." S. T. Turner, El Paso.

### OBSTETRICS AND DISEASES OF CHILDREN.

1. Address of Chairman. J. M. Frazier, Belton.

*Special Assignment.*

2. "Importance of Early Recognition and Prompt Rectification of Deviations from the Normal Mechanical Evolutions of Labor."

J. F. Y. Paine, Galveston. Discussion to be opened by J. E. Gilcreest, Gainesville.

3. "The Management of an Uncomplicated Case of Labor by the General Practitioner." W. H. Monday, Terrell.

4. "The Management of the Third Stage of Labor and of Hemorrhage." B. F. Kingsley, San Antonio.

5. "Eclampsia." Aug. D. Ferguson, Frost.

6. "Summer Complaints of Children." Aug. D. Ferguson, Frost.

### SURGERY.

1. Report of Chairman. A. C. Scott, Temple.

2. Address and Clinic. (By invitation.) James P. Tuttle, New York City.

3. "Fever of the Puerperal State from a Surgical Standpoint." Emory Lanphear, St. Louis, Mo. (By invitation.)

4. "Holocain as a Local Anesthetic." (By invitation.) Frank T. Smith, Chattanooga, Tenn.

5. "Injuries to the Eye." John O. McReynolds, Dallas.

6. "Prevention and Treatment of Shock in Laparotomies." F. D. Thompson, Fort Worth.

7. "Interscapulo Thoracic Amputation." James E. Thompson, Galveston.

8. "Chronic Contraction of the Prostatic Fibers Encircling the Vesical Neck—Report of a Case." J. B. Shelmire, Dallas.

9. "Operative Treatment of Enlarged Prostate." Bacon Saunders, Fort Worth.

10. "Report of Some Surgical Operations." J. R. Stuart, Houston.

11. "Cranial Injuries—Report of Two Cases." F. B. Shields, Victoria.

### MEDICAL JURISPRUDENCE.

1. Report of Chairman—"Some Problems of Texas Insane Asylums." Marvin L. Graves, San Antonio.

2. "The Insane and Hospital Management." John T. Turner, Terrell.

3. "The Criminal Insane." G. H. Moody, San Antonio.

4. "School Life and Insanity." J. S. Lankford, San Antonio.

5. "The Physician as an Expert Witness." R. B. Sellers, San Antonio.

6. "Subconscious Homicide and Suicide, their Physiological Psycology." Charles P. Bancroft, Concord, N. H. (By invitation.)

### STATE MEDICINE AND PUBLIC HYGIENE.

1. Chairman's Address. W. S. Carter, Galveston.

2. "The Paramount Duty Texas Owes to Her People." F. E. Daniel, Austin.

3. "The Influence of Flies in the Spread of Typhoid Fever." C. E. D. Lord, Galveston.

*Symposium on the Spread and Prevention of Tuberculosis.*

4. "The Inheritance of Tuberculosis." A. J. Smith, Galveston.

5. "Relation of Bovine to Human Tuberculosis." Wm. Gammon, Galveston.

6. "Surgical Tuberculosis." Wm. Keiller, Galveston.

7. "Tuberculosis in our Public Institutions." John S. Turner, Terrell.

8. "The Introduction and Development of Tuberculosis in San Antonio." Frank Paschal, San Antonio.

9. "Tuberculosis in El Paso." J. A. Rawlings, El Paso.

10. "Prevention of Tuberculosis in Private Practice." J. W. McLaughlin, Galveston.

11. "Public Control of Tuberculosis." Lawrence Flick, Philadelphia. (By invitation.)

### GYNECOLOGY.

1. Chairman's Address. J. T. Feild, Fort Worth.

*Special Assignment.*

2. "Operation for Complete Laceration of the Perineum without Leaving Stitches in the Bowel." C. E. Cantrell, Greenville. To open discussion, I. N. Suttle, Corsicana.

3. "Mammary Substance in Uterine Fibroids." H. W. Crouse, Victoria.

4. "Dysmenorrhea." M. M. Pool, El Campo.

5. "Ectopic Gestation." W. E. Fowler, Huntsville.

6. "Conservative Treatment of Uterine Displacement." J. M. Inge, Denton.

7. "The Relation the Uterus Bears to the Mind." J. C. Carlton, Bonham.

8. "Retrodisplacements of the Uterus." A. L. Hatchcock, Palestine.

9. "Genital Reflexes." John Rodman, Waxahachie.

### OPHTHALMOLOGY, ETC.

1. Chairman's Address. Robt. E. Moss, San Antonio.
2. "Operative Treatment for Glaucoma." W. A. Harper, Austin.
3. "Radical Cure of Tonsilitis." Frank D. Boyd, Fort Worth.
4. "Nose Washing." Clarence Warfield, San Antonio.
5. "Nature and Treatment of Pterygia." John O. McReynolds, Dallas.
6. "Laryngeal Diphtheria." E. D. Capps, Fort Worth.
7. "Recovery from Tetanus Following Gunshot Wound in Face and Orbit." H. L. Hilgartner, Austin.
8. "Some Abused Eyes." R. F. Miller, Sherman.
9. "Primary Ephithelioma of the Cornea." Hy. Redmond, Corpus Christi.

### PATHOLOGY.

1. "Chairman's Address. A. J. Smith, Galveston.
2. "Pharyngeal False Membranes in Croupus Pneumonia." J. M. Frazier, Belton.
3. "Persistence of Foramen Ovale in Adult Hearts." H. B. Dechard, Galveston.
4. "Skiagraphy of Renal Circulation." W. Keiller, Galveston.
5. "Period of Latency in Malaria." John T. Moore, Galveston.
6. "Renal Adenomata." J. E. Thompson, Galveston.

The indications are favorable for a large attendance at Dallas. The presence and co-operation of all regular and reputable physicians in the State is earnestly solicited.

The scientific program is sufficiently varied to suit the taste of all. The most important matter ever presented to the Association will be introduced for consideration and action, viz.: Revision of our organic law. The services of an expert medical stenographer, Mr. Samuel Bennett, of New York, has been secured for the occasion.

Fraternally,

H. A. West, Secretary.

